# The Contribution of *VKORC1* and *CYP2C9 *Genetic Polymorphisms and Patients’ Demographic Characteristics with Warfarin Maintenance Doses: A Suggested Warfarin Dosing Algorithm

**DOI:** 10.22037/ijpr.2020.1101116

**Published:** 2020

**Authors:** Esmat Khaleqsefat, Mohammad Khalaj-Kondori, Mortaza Jabarpour Bonyadi, Hamid Soraya, Behnam Askari

**Affiliations:** a *Department of Animal Biology, Faculty of Natural Sciences, University of Tabriz, Tabriz, Iran. *; b *Department of Pharmacology, Faculty of Pharmacy, Urmia University of Medical Sciences, Urmia, Iran. *; c *Department of Cardiovascular Surgery, Seyyed-al-Shohada Heart Center, Urmia University of Medical Sciences, Urmia, Iran.*

**Keywords:** VKORC1, CYP2C9, Genetic Polymorphism, Warfarin, Demographic Characteristics

## Abstract

The requirement of varying doses of warfarin for different individuals can be explained by environmental and genetic factors. We evaluated the frequency of vitamin K epoxide reductase complex subunit 1 (*VKORC1*) and cytochrome P450 2C9 (*CYP2C9*) variants together with patientdemographic characteristics and investigated their association with warfarin dose requirement with the objective to suggest a warfarin dosing algorithm. In this study, 185 patients with heart valve replacement from West Azerbaijan, Iran were genotyped for *VKORC1* (-1639 G>A) and *CYP2C9 *(*2 and *3 alleles) by PCR-RFLP. Multiple linear regression was performed to create a new warfarin dosing algorithm. The frequency of variants in studied subjects was 12% for CYP2C9 *2, 25.8% for CYP2C9 *3, and 60% for -1639A. The patients who carried the A allele at position -1639 *VKORC1* and the variants CYP2C9 *2 and *3 required a significantly lower daily mean warfarin dosage (*P *= 0.001). Statistical analysis also indicated a significant relationship between the daily maintenance dose of warfarin with age and blood pressure among the studied patients’ cohort *(P *< 0.001). This study showed that in the heart valve replacement patients considering *VKORC1* and *CYP2C9 *polymorphisms beside demographic characteristics such as age will be helpful in pre-treatment dosing of warfarin which in turn reduces the complications associated with inappropriate warfarin dosing.

## Introduction

The patients who have undergone heart valve replacement need warfarin, an anticoagulant, for the rest of their lives (1). However, maintaining the therapeutic doses of warfarin is still problematic especially in the initial stage of warfarin therapy, because of its narrow therapeutic index and large inter-individual variability in the patient’s response. A sub-therapeutic dose might not prevent clot formation and stroke, while a high dose might leads to bleeding (2). Thus, the appropriate dose of warfarin must be personalized for each patient. The criterion used for response to this drug is the prothrombin ratio (PR) expressed as international normalized ratio (INR) and measured by the clotting tendency of blood (3). Warfarin dose requirement is determined by a number of different factors, with *VKORC1* genotype being the major constituent (by 33%) while *CYP2C9 *genotype (by 22%) together with age, weight, and gender (by 12%) also contribute (4, 5). 

Vitamin K epoxide reductase complex (*VKORC1*) which encodes the VKOR enzyme is located on chromosome 16 p 11.2 (6). The VKOR enzyme is essential for the coagulation process and its inhibition by warfarin leads to perturbation of blood coagulation (7). The genetic polymorphism -1639 G>A is located in the promoter region of the *VKORC1* gene at a potential E box (CA/GNNTG). It has been shown that this position is an important element for mediating the transcription of *VKORC1* and that it will change the promoter activity when adenine substitutes for guanine at the position (8). Due to the -1639 G>A polymorphism, the qualitative and quantitative expression of *VKORC1* and subsequently of warfarin dose requirement could be significantly influenced (9, 10).

Warfarin is a racemic mixture of S- and R- isomers of which S-warfarin has considerably more anticoagulant activity (11). The *CYP2C9 *gene located on the forward direction of chromosome 10, encodes enzyme which involves in the clearance of various therapeutic agents including S-warfarin (12). The allelic variants CYP2C9 *2 (rs 1799853, c. 430 C>T, p. Arg144Cys) and *3 (rs 1057910, c. 1075 A>C, p. Ile359Leu), are most common functionally significant coding SNPs that affect enzyme activities and cause impaired metabolic capacity of *CYP2C9 *enzyme. The patients with one or two of these variants require lower dose of warfarin and encounter higher risk of bleeding compared with the patients carrying wild type (*1) allele (13).

Different allelic frequencies have been shown for* VKORC1 *-1639 G>A and *CYP2C9 *variants in various ethnic groups. Iran is a country with different ethnic populations including Turks, Arabs, Baloches, and Kurds, which may have different genetic, social and environmental backgrounds. However, there are limited data on the genetic polymorphisms related to warfarin metabolism and action for the Iranian population (14). Hence, this study for the first time aimed to assess the effect of* VKORC1* -1639 G>A and *CYP2C9 *variants together with demographical characteristics of patients on warfarin doses requirement among patients mostly with heart valve replacement in West Azerbaijan, Iran. 

## Experimental

*Subjects and Criteria*

This study was performed between February 2016 and March 2017 on a total of 185 patients aged 25-86 mostly with heart valve replacement. The patients who received a maintenance dose less than 1.5 mg/day, were considered as sensitive to warfarin. Patients on more than 7.5 mg/day were considered as warfarin resistant. The patients with intermediate dosage of warfarin (1.5 to 7.5 mg/day) were considered as control population (15). The patients who had stable doses and international normalized ratio (INRs) between the ranges of 2.5- 3.5 within the last 4 weeks were included in the study. The exclusion criteria were suffering from cancer, liver, and kidney diseases. All procedures performed in this study were approved by the Scientific and Ethical Committee of Urmia University of Medical Sciences (Ir.umsu.rec.1394.282).

A volume of 2 mL of peripheral blood obtained from each patient was transferred to EDTA- containing tubes in order to prevent clotting and stored at -20 °C prior to the experiments. The prothrombin time- international normalized ratio (PT-INR) test was used to determine the clotting tendency of the blood samples in the measurement of warfarin dosage. Questionnaires were designed for the demographic characteristics of the patients including sex, age, weight, height, ethnicity, type of heart valve, diabetes, blood pressure, and smoking.

*Genotyping of VKORC1 and CYP2C9 alleles*

Genomic DNA was extracted from individual blood samples employing DNA extraction mini kit (YTA Company, Iran) according to the manufacturer’s instructions. To detect polymorphism -1639 G>A in *VKORC1* a polymerase chain reaction-restriction fragment length polymorphism (PCR-RFLP) analysis was carried out according to Sconce *et al.* (2005) (3). To detect CYP2C9 *2 and CYP2C9 *3 polymorphic alleles, a polymerase chain reaction-restriction fragment length polymorphism (PCR-RFLP) analysis was performed as described by Daly *et al.* (2006) (16). 

*Statistical analysis*

Mean ± standard deviation (SD) along with median interquartile range (IQR) of warfarin doses were calculated in demographic, biologic, and clinical variables groups. The Pearson correlation test was used to examine the association of age, Height, weight, and BMI with warfarin dose. Significance differences in daily maintenance dose of warfarin between different genotype groups were evaluated by independent samples t-test and one-way analysis of variance (ANOVA). The normality assumption of warfarin dose was evaluated by Shapiro-Wilk test. If needed the square root transformation was done to normalize warfarin dose distribution and calculate the correct *p*-value in comparisons. The genotype distributions for the studied SNPs were calculated and checked to be in Hardy-Weinberg equilibrium (HWE) using Chi-square goodness-of-fit test with Degrees of freedom (df) of one. Box plot analysis was performed to graphically display the dispersion and skewness of warfarin dose among the genotype groups. The backward stepwise linear regression analysis was used to model the relationships of the patient’s demographic variables and genotypes with daily warfarin dose requirements. At the first step of backward regression method all of the studied variables including; age (years), ethnicity (Turk/others), hypertension, *VKORC1* and *CYP2C9 *genotypes, BMI (kg/m^2^), sex (female/male) and smoking were included in the model. At the next steps the variables with weakest none-significant effect were excluded and ultimately a novel warfarin dosing algorithm was presented. All of calculations and statistical analysis were done by IMB SPSS Statistics 23 (IBM SPSS Inc. Chicago, IL). The statistical level of significance was set at *P* < 0.05.

## Results

Among 200 patients mostly with heart valve replacement, 185 individuals (112 females and 73 males; mean age 54.7 ± 14) who met the inclusion criteria were included in the study. Distribution of the daily dosage of warfarin was 0.28 to 12.10 mg/day among all the patients while the mean daily dose of warfarin was 4.26 ± 2.43 mg/day. The descriptive statistics of the patients’ demographic, biologic, and clinical variables are shown in Table 1. 

The *VKORC1* (rs 9923231, -1639 G>A) polymorphism, genotype frequencies were 20.5%, 40.5%, and 38.9% for GG, GA, and AA genotypes, respectively. The allelic frequency for *VKORC1* -1639 A was 60%. In the studied population the CYP2C9 *2 mutant allele frequency was found to be 12.6%. Out of 170 patients, 39 were heterozygous (CT) (22.9%) and 2 were homozygous (TT) (1.2%). The CYP2C9 *3 mutant allele frequency was found to be 25.8%. Out of 170 individuals, 82 were heterozygous (AC) (48.2%) and 3 were homozygous (CC) (1.8%). Overall genotype frequency for* CYP2C9 *was determined to be CYP2C9 *1/*1 (35.7%), *1/*2 (16.2%), *1/*3 (35.1%), *2/*2 (1.6%), *2/*3 (9.2%), and *3/*3 (2.2%). No significant deviation from Hardy Weinberg equilibrium was observed for *CYP2C9 *variants (*P *> 0.05).

The results of the mean comparison among the studied patients’ cohort revealed that the mean of warfarin dose had not significant difference with sex, heart valve type, smoke, diabetes, and hypertension (*P *> 0.05). The patients who consumed alcohol with the mean warfarin dose of 6.39 ± 2.45 mg/day (*vs.* non-alcohol consumers) had significantly higher and the Turkish patients with the mean warfarin dose of 4.16 ± 2.52 mg/day (*vs.* other ethnicities) had significantly lower warfarin dose requirement (*P* > 0.05). The Pearson correlation result showed significantly negative association between age and warfarin dose (r = -0.371, *P* < 0.001) (Table 2).

**Figure 1:**
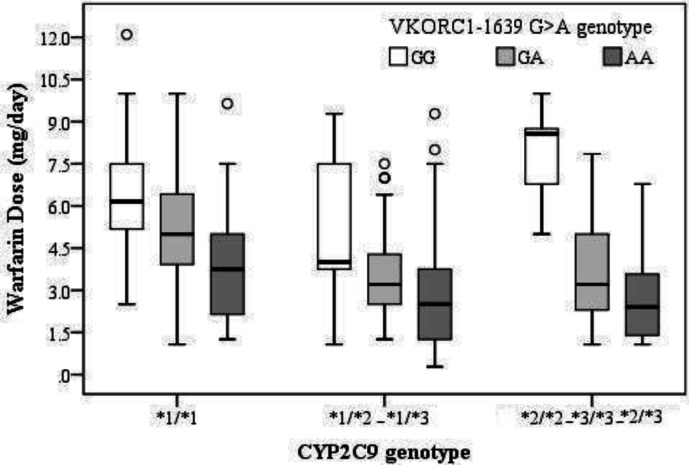
Boxplot showing different genotype group of *CYP2C9* and *VKORC1* -1639 against daily warfarin dosage (mg/d). Boxes indicate the median and interquartile ranges. Vertical lines above and below boxes indicate the minimum and maximum values

 Comparison of the mean warfarin doses requirements among *VKORC1* -1639 genotypes revealed significant differences between the genotypes. Mean warfarin dose requirement was obtained 6.13 ± 2.59 mg/day for people with genotype GG, 4.15 ± 1.92 mg/day for genotype GA and 3.37 ± 2.29 mg/day for the genotype AA. Pairwise comparisons of mean warfarin dose requirements among the patients with GG, GA, and AA genotypes indicated significant differences (*P* < 0.001 for GG vs GA and GG vs AA, *P* = 0.02 for GA *vs.* AA). Also, the mean of warfarin dose requirements significantly differed among *CYP2C9 *genotypes. Pairwise comparisons of warfarin dose requirements across the patients with genotypes of ^*^1/^*^1 (5.10 ± 2.40 mg/day), ^*^1/^*^2, ^*^1/^*^3 (3.65 ± 2.18 mg/day), and ^*^2/^*^2, ^*^2/^*^3, ^*^3/^*^3 patients (4.32 ± 2.82 mg/day) indicated that the mean warfarin dose requirement of the people with genotype ^*^1/^*^1 significantly differed from that of the studied cohort with the genotype ^*^1/^*^2, ^*^1/^*^3 (*P *< 0.001). However, the comparisons among ^*^1/^*^1 vs. ^*^2/^*^2, ^*^2/^*^3,^ *^3/^*^3 (*P *= 0.22) and^*^1/^*^2, ^*^1/^*^3 *vs.* ^*^2/^*^2, ^*^2/^*^3,^ *^3/^*^3 (*P *= 0.54) showed no significant differences (Table 3). The simultaneous effect of the variant alleles of *VKORC1* -1639 G>A and *CYP2C9 *is shown in Figure 1.

Final regression model results of warfarin dose requirement showed significant effects of age, hypertension, and genotype on warfarin dose (*P *< 0.001) (Table 4). The negative impact of age was significant on warfarin dose (*β* = -0.02, *P *< 0.001). Hypertensive patients had a higher warfarin dose requirement (*β* = 0.27, *P *< 0.001). The examination of the *VKORC1* -1639 G>A genotype effect indicates that the patients with AA (β = -0.62, *P *< 0.001) and GA genotypes (*β* =-0.43, *P *= 0.001) had lower warfarin dose requirement compared to GG genotype. The patients with *1/*2, *1/*3 (*β* = -0.34, *P* < 0.001) and *2/*3, *2/*3 (*β *= -0.28, *P* = 0.017) of *CYP2C9 *genotype had lower warfarin dose requirement compared to *1/*1 genotype. The stepwise evaluation of the effect of the patients’ demographic, biologic, and clinical variables and genotypes indicated that the order of variables elimination from regression models, according to weakest impact, were as follows: sex, smoking, alcohol, diabetes, ethnicity, BMI and in the final step the valve type was excluded from the model.

## Discussion

Optimizing the appropriate dose of warfarin for each individual based on genetic and environmental factors, especially in the initial stage of its usage, will lead to a reduction in the side effects of this drug and in turn decreased healthcare costs for the patients and hospitals (2, 17). The aim of the current study was to investigate the effect of -1639 G>A *VKORC1* and *CYP2C9 *polymorphisms together with non-genetic factors that might contribute warfarin-dose variability, with the view of developing an individualized dosing regimenin North-western of Iran.

The association of *VKORC1* polymorphism with warfarin dose requirement in this study was in agreement with what was reported in other populations such as United Kingdom (3), United States (18), Turkey (19), and China (20). The role of *VKORC1* polymorphisms in warfarin maintenance dose was identified by D’ Andrea *et al. *(21). Yuan and colleagues in 2005 have reported the association of -1639A allele with low warfarin dose requirement (8). Rieder *et al. *have introduced haplotypes for *VKORC1* on the basis of a panel of SNPs and concluded that some polymorphisms are informative about warfarin dose among European–Americans (22). Interestingly, while *VKORC1* -1639 G>A is important in the patients who are sensitive or resistant to warfarin, different genotype frequencies were reported from seven East Asian countries (Taiwan, India, Indonesia, Philippines, Thailand, Vietnam and China). For example, AA and GG genotype frequencies were 67% and 6% in China’s population but 7% and 80% in India, respectively (23). 

The association of *CYP2C9 *variants with warfarin dose requirement also has been reported in 1999 for the first time (24). The presence of CYP2C9 *2 and *3 alleles emphasizes the fact that a drug *e.g.* warfarin will be metabolized more slowly and individuals carrying these alleles will be more likely at the risk of bleeding and severe drug poisoning. Different allelic frequencies have been reported for CYP2C9 *2 and *3 in different racial groups. The allelic frequencies of CYP2C9 *2 and *3 in our study were 12.5% and 25.8%, respectively. But the frequency of these alleles has been reported 9.1% and 10%, respectively in the northwest of Iran. In the investigation conducted on 120 individuals in northeast of Iran 9.1% for CYP2C9 *2 and 10% for *3 were reported which showed lower variant allele frequencies in comparison to our research (25). Our studied region is located in northwest of Iran near to the border of Turkey and the studied subjects are comprised of mostly the Turks population. So, we compared the *CYP2C9 *allelic frequency results in our study with those in Turkey and observed similarity to those of Yildirim *et al.* (17% for *2 and 26% for *3) (5) and Ozer *et al.* (2010) (13% for *2 and 15% for *3) in the Turkish population (26). Our frequency result was also different from European population (12.5% CYP2C9 *2 and 8.5% CYP2C9 *3) (27).

Studies have also revealed that several factors affect the variability in warfarin dose, including age, body size, vitamin K intake, interacting medications, and genetic variants (28). This study showed that the ethnicity is an important factor in distribution of genetic polymorphisms. Previous studies also proved the role of ethnicity in *CYP2C9 *and *VKORC1* variant frequency and in turn the amount of warfarin was required among Asian, European, and the other ethnicities. The role of age also has been investigated and the negative correlation between age and warfarin clearance was reported (5). The older patients require a lower dose of warfarin possibly because their liver mass and subsequently content of liver VKOR is decreased, so they become more warfarin sensitive. Park *et al.* (2013) demonstrated the relation between warfarin dosage and age, body mass index plus genetic variants (29). However, the current warfarin dosing algorithms do not incorporate genetic and environmental factors that affect warfarin-dose requirements. Knowledge of the extent to which these factors affect anticoagulation response along with genotyping methods could help in the prediction of a more individualized loading and maintenance warfarin dose for a safer anticoagulation therapy (30). In this research we have found out that age, ethnicity and type of heart disease beside genotype affect the amount of warfarin required in our patient group.

In the multi-ethnic population of Iran, genetic diversity, and different allelic and genotypic prevalence is predictable. It is essential to investigate the distribution patterns of genetic variants in different region of country to develop a proper dosing algorithm of warfarin and better understanding of the relationship between *VKORC1* and *CYP2C9* variants with sensitivity to warfarin. Determining the genotypes and changes which may influence enzyme expression allows us to truly personalize warfarin dosing.

**Table 1:** Descriptive statistics of demographic, biologic and clinical variables among study population (n=185).

**Categorical Variables**	**Categories**	**n**	**%**
Sex	Male	73	39.5
	Female	112	60.5
Heart Valve Type	AVR	41	25.6
	MVR	57	35.6
	AVR MVR	42	26.3
	AF	13	8.1
	Other	7	4.4
Smoke	Positive	17	9.2
	Negative	168	90.8
Alcohol	Positive	6	3.2
	Negative	179	96.8
Diabetes	Positive	22	11.9
	Negative	163	88.1
Hypertension	Positive	57	30.8
	Negative	128	69.2
Ethnicity	Turk	157	84.9
	Other	28	15.1
*VKORC1*-1639	GG	38	20.5
	GA	75	40.5
	AA	72	38.9
*CYP2C9 *	*1/*1	66	35.7
	*1/*2, *1/*3	95	51.4
	*2/*2, *2/*3, *3/*3	24	13.0
Warfarin Dose	0-1.5	30	16.2
	1.6-7.4	125	67.6
	7.5-12	30	16.2
**Continuous Variables**	**Mean ± SD**		**Range**
Age (year)	54.72 ± 14.04		20 – 84
Height (cm)	72.65 ± 14.80		41 – 123
Weight (kg)	163.22 ± 10.19		120 – 186
BMI (kg/m^2^)	27.28 ± 5.36		14.53 – 48.59
Warfarin dose (mg/day)	4.25 ± 2.43		0.28 – 12.10

**Table 2:** The relationship of demographic, biologic and clinical variables to the warfarin maintenance dose. The mean and median for warfarin dose were reported in variable groups. (n=185).

**Categorical Variables**	**Categories**	**Mean ± SD**	**Median (IQR)**	***P*** **-value**
Gender	Male	4.01 ± 2.31	3.57 (2.50)	0.32
	Female	4.42 ± 2.50	3.92 (3.91)	
Valve Type	AVR	4.99 ± 2.77	3.90 (4.23)	0.18
	MVR	4.00 ± 2.04	3.75 (2.68)	
	AVR MVR	4.90 ± 2.55	5.00 (4.13)	
	AF	3.90 ± 2.04	3.90 (3.21)	
	Other	3.70 ± 2.06	3.57 (2.70)	
Smoke	positive	4.34 ± 2.74	3.21 (3.80)	0.88
	negative	4.25 ± 2.40	3.75 (3.12)	
Alcohol	positive	6.39 ± 2.45	6.68 (4.66)	0.03
	negative	4.18 ± 2.40	3.70 (2.85)	
Diabetes	positive	5.23 ± 2.97	4.64 (4.63)	0.07
	negative	4.12 ± 2.32	3.70 (2.85)	
Hypertension	positive	4.61 ± 2.68	4.28 (4.24)	0.23
	negative	4.10 ± 2.30	3.57 (2.85)	
Race	Turk	4.16 ± 2.52	3.57 (3.02)	0.03
	Other	4.78 ± 1.77	5.00 (3.08)	
**Continuous Variables**	**R** **(Pearson Correlation)**		***P*** **-value**	
Age (year)	-0.371		<0.001	
Height (cm)	-0.012		0.88	
Weight (kg)	-0.086		0.30	
BMI (kg/m^2^)	0.089		0.29	

IQR: Inter-Quartile Range; BMI: Body Mass Index.

**Table 3:** The relationship among *VKORC1*-1639 G>A and *CYP2C9 *genotypes with warfarin maintenance dose (n=185).

**Categorical Variables**	**Categories**	**Mean ± SD**	**Median (IQR)**		***P*** **-value**
*VKORC1*-1639	GG	^a^6.13 ± 2.59		5.53 (4.40)	<0.001
	GA	^b^4.15 ± 1.92		3.57 (2.50)	
	AA	^c^3.37 ± 2.29		2.50 (3.40)	
*CYP2C9 *	*1/*1	^a^5.10 ± 2.40		5.00 (3.68)	0.001
	*1/*2- *1/*3	^b^3.65 ± 2.18		3.21 (2.70)	
	*2/*2- *3/*3- *2/*3	^ab^4.32 ± 2.82		3.57 (5.32)	

^a, b^ and ^ab^ indicate that the groups with different word have a significant mean difference due to Tukey-HSD pairwise comparison.

**Table 4:** Linear regression model of warfarin daily dose requirement affected by age, blood pressure and genotype

**independent variables**	**Categories**	**Beta**	**Std. error**	***P*** **-value** **(parameters)**	***P*** **-value** **(between group)**
Intercept		3.42	0.16	< 0.001	< 0.001
Age		-0.016	0.003	< 0.001	< 0.001
Blood Pressure					< 0.001
	Positive	0.270	0.079	< 0.001	
*VKORC1*-1639					< 0.001
	AA *vs* GG	-0.624	0.096	< 0.001	
	GA *vs* GG	-0.427	0.095	0.001	
*CYP2C9*					< 0.001
	*1/*2-*1/*3 vs. *1/*1	-0.337	0.076	<0.001	
	*2/*2- *2/*3-*3/*3 *vs*. *1/*1	-0.275	0.114	0.017	

Square root transformation was done on warfarin dose value.

R^2^ for model equal 39%.

Regression equation: SQRT (Dose) = 3.42 – 0.02 (age) + 0.27 (hypertensive) - 0.62 (*VKORC1*-1639 genotype = AA) - 0.43 (*VKORC1*-1639 genotype = GA) - 0.34 (*CYP2C9 *genotype = (*1/*2 or *1/*3) - 0.28 (*CYP2C9 *genotype = (*2/*2 or *3/*3 or *2/*3)

*CYP2C9 *genotype input *CYP* (*1/*1) as 0, (*1/*2 or *1/*3) as 1 and (*2/*2 or *3/*3 or *2/*3) as 2; *VKORC1* -1639 G>A genotypes input 1 for GG, 2 for GA and 3 AA; Age input in years; Blood pressure 1 for positive, 0 for negative.

## Conclusion

In conclusion, the results of the present study showed that prescribing an appropriate dosing regimen of warfarin in accordance with the patient’s demographic and pharmacogenetic data is on the benefit of the patients treated with warfarin. This study demonstrated that the patients with* VKORC1* -1639 A and CYP2C9 *2, *3 alleles would require lower dose of warfarin and are mainly considered as warfarin sensitive. As carrying the variant alleles *CYP2C9 *and *VKORC1* A allele cause sensitivity to warfarin and the prevalence of these variants is high in the study population, considering this fact before prescribing warfarin is important.
